# A Rare Presentation of Hydatid Cyst: A Case Report of Uncommon Localization in the Pelvic Region and a Review of Current Literature

**DOI:** 10.7759/cureus.60312

**Published:** 2024-05-14

**Authors:** Mehmet Gurcan, Rifat Burak Ergul, Enes Degirmenci, Murat Dursun, Ateş Kadıoğlu

**Affiliations:** 1 Urology, Istanbul University School of Medicine, Istanbul, TUR

**Keywords:** genitourinary tract, exceptional localization, retrovesical, echinococcus granulosus, cyst hydatid

## Abstract

Hydatid cyst disease, caused by the *Echinococcus granulosus* tapeworm larval form, typically affects the liver, lungs, and genitourinary tract. This case report details an unusual manifestation of hydatid cysts in a 62-year-old male with a history of retrovesical hydatid cyst surgery. The patient presented with pain at the root of the penis, and subsequent imaging revealed cysts in the suprapubic, pubic bone, and left pectineus muscle regions. Despite the challenges posed by the COVID-19 pandemic, the patient underwent surgical excision in 2023. The discussion emphasizes the rarity of such localized cysts, diagnostic imaging techniques, and the necessity of surgical intervention. The postoperative period involved a course of albendazole. While rectovesical hydatid cysts are uncommon, this case underscores the importance of considering them in the differential diagnosis of masses, particularly in endemic regions. Surgical excision remains the primary treatment for symptomatic hydatid cysts.

## Introduction

Hydatid cyst disease is an orally transmitted parasitosis that occurs when the larval form of the *Echinococcus granulosus* tapeworm penetrates the intestinal mucosa and reaches the internal organs through blood and lymphatic flow [1]. It is endemic in North Africa, Spain, Greece, Turkey, Portugal, the Middle East, Australia, New Zealand, South America, the Baltic regions, and the Philippines [2]. The most common sites of infection are the liver (75%), lung (15%), brain (2-4%), and genitourinary tract (2-3%) [3]. In this case report, we aimed to manage a case of hydatid cysts localized in unusual regions.

## Case presentation

A 62-year-old male patient with no additional disease other than known hypertension and diabetes mellitus was operated on at another center for a retrovesical hydatid cyst in 2011. During the surgery, the cyst was ruptured, and the cyst material was excreted into the abdomen. It was successfully managed with a vacuum-assisted closure. The patient was admitted to our department with a complaint of pain at the root of the penis in 2017. In the physical examination, swelling was inspected in the left suprapubic region, and an immobile mass lesion was palpated. The hydatid hemagglutination test yielded positive results. In an MRI performed in 2019, a lesion measuring 96x80x71 mm, consistent with type III cystic hydatidosis, was identified between the symphysis pubis and the corpus cavernosum superior to the penis root. Similar cystic formations were noted in the symphysis pubis, the right pubic bone adjacent to the symphysis pubis, the entire left pubic bone, and in the left iliac chain neighborhood, measuring approximately 22x13 mm in the left obturator region, indicative of type IV hydatid cysts. Lesions measuring 5.5x3 cm and 2.5x2.5 cm were also found in the left pectineus muscle.

**Figure 1:**
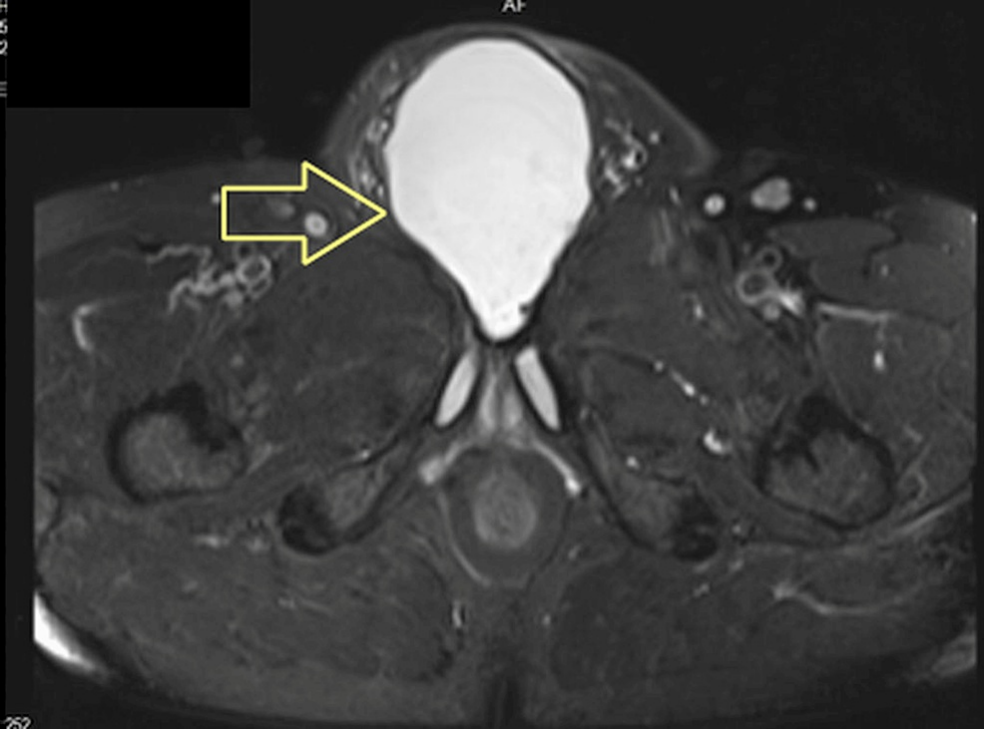
The cyst between the symphysis pubis and the penis root

**Figure 2:**
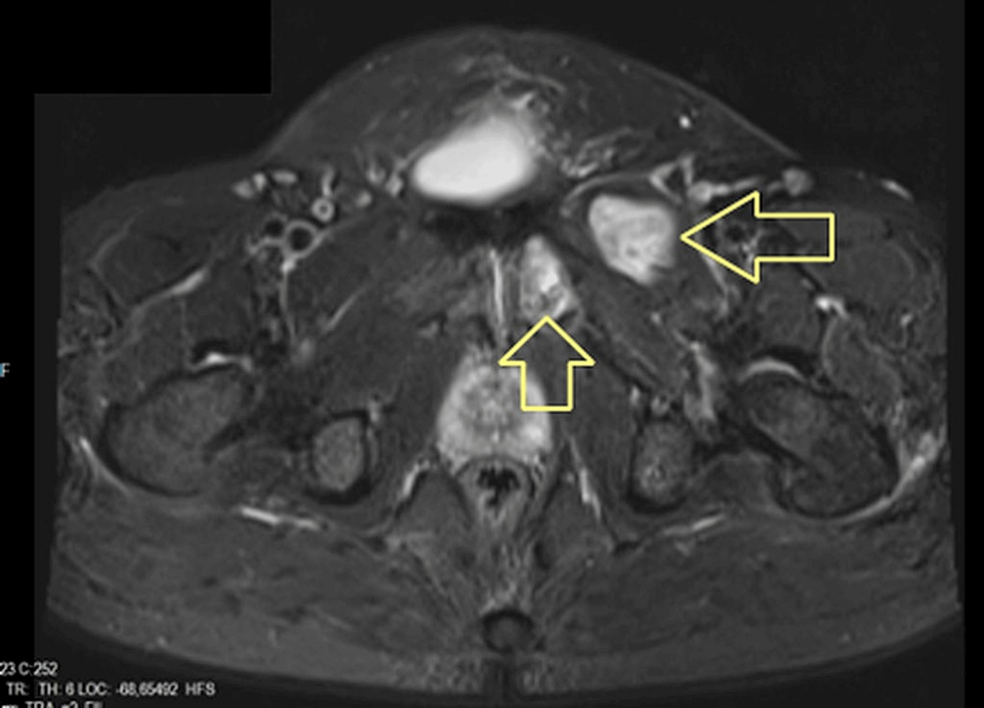
The cyst within the left pectineus muscle

**Figure 3:**
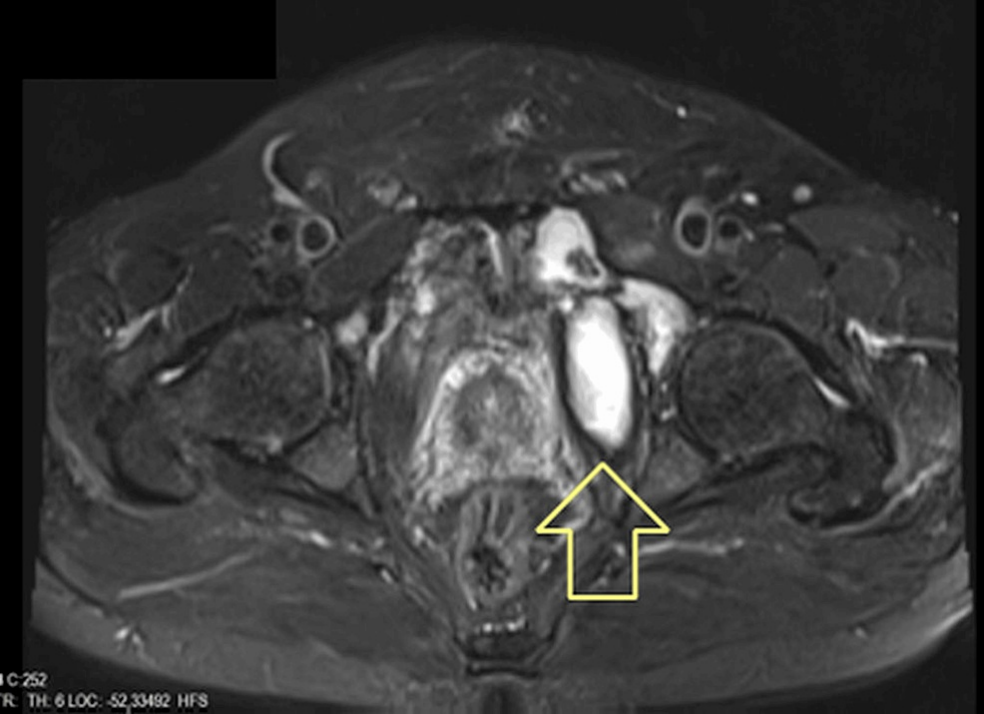
The rectovesical cyst

Due to the COVID-19 pandemic, the patient was not followed up until December 2022, when he returned to our clinic with complaints of suprapubic pain and swelling. A subsequent MRI in December 2022 revealed a cystic lesion measuring 121x71x75 mm in size in the pubic region. It extended posteriorly to the vicinity of the symphysis pubis superior to the penis root and was adjacent to the corpus cavernosum caudally. This was considered compatible with a type 2 hydatid cyst in the pubic region. Additionally, a lesion compatible with hydatid bone involvement in the left obturator site, approximately 27x59 mm, and nodular lesions consistent with type IV hydatid cyst measuring 44x34 mm were detected in the left pectineus muscle (Figures 1-3). On January 9, 2023, the patient underwent inguinal hydatid cyst excision and left pectineal muscle excision surgery. A decision was made for follow-up on hydatid cysts localized to the retrovesical and pubic bones due to their benign nature, the absence of gait symptoms, and the potential morbidities associated with further surgical intervention. There are no specific recommendations for the medical treatment of rectovesical hydatid cysts in the literature. In this particular instance, surgical intervention followed a 14-day course of albendazole at a dosage of 2x400 mg orally. Subsequently, albendazole treatment at a dose of 2x400 mg was continued for an additional 4 weeks in the postoperative period.

## Discussion

Hydatid cyst disease is a zoonotic and parasitic infection caused by the larval stage of *Echinococcus granulosus*. Serology can be utilized for diagnosis [4]. Intra-abdominal or pelvic hydatid cysts often result from secondary traumatic, spontaneous, or iatrogenic perforation of hydatid cysts located in another organ [5]. Rectovesical hydatid cysts account for 0.1-0.5% of all cases of hydatid cysts. In a review article discussing rare localizations of hydatid cysts, presentations in the skeleton (0.2-3%), peritoneum (2-5.2%), spleen (0.9-8%), kidney (0.4-3.7%), brain (0.4-1%), cardiac muscle (0.02-1.1%), and subcutaneous (1.6%) were reported [6].

Cysts are mostly asymptomatic, and patients are often examined following clinical evidence of a mass or unclear symptoms. In cases where a hydatid cyst is located in the retrovesical region, it can cause symptoms such as flank pain, palpable mass, urinary retention, obstruction, and urgency [7]. However, in our patient, there were no such symptoms; the major complaint was pain in the root of the penis.

Ultrasonography, CT, and MRI are useful imaging techniques for diagnosing unusually localized hydatid cysts [8]. Hydatid cysts can be categorized into five types according to the imaging classification proposed by Gharbi et al. [9]. Types II, III, and V typically present no diagnostic challenges. A heterogeneous hyperechoic well-circumscribed retrovesical lesion may resemble a type IV hydatid cyst, a pelvic abscess, or a pelvic tumor [9].

Postoperative treatment should involve the use of either albendazole or mebendazole. Administering albendazole preoperatively at a dosage of 10 mg/kg/day for one month effectively eliminates a majority of the protoscoleces within the hepatic hydatid cyst [10]. There are no specific recommendations for the medical treatment of rectovesical hydatid cysts in the literature. In this specific case, surgical intervention was preceded by a 14-day course of albendazole at a dosage of 2x400 mg orally. Following surgery, albendazole treatment at the same dosage was continued for an additional 4 weeks in the postoperative period.

The gold standard treatment for cyst hydatid is the total excision of the cyst, preventing its rupture and spillage. Care should be taken for anaphylactic shock if the cyst ruptures. In cases of rupture, aspiration and the use of a hypertonic solution are required. In our case, the necessary surgery included the en bloc removal of the hydatid cyst close to the crus of the penis. The cyst was incised, and 20% saline was used around the incision. The cyst wall was opened with scissors, and the cystic fluid inside was evacuated with an aspirator. Finally, the cyst sac was washed with a 20% saline solution.

## Conclusions

While rectovesical hydatid cysts are uncommon, it is crucial to consider them in the differential diagnosis of rectovesical masses, especially in regions where they are endemic. Utilizing clinical history, preoperative radiological assessments, and serological tests can help in accurately diagnosing this condition. A surgical approach is the most appropriate treatment option for patients with hydatid cysts that cause compression symptoms.
